# Effort–reward imbalance at work and health: Review and critical appraisal of three decades of research

**DOI:** 10.5271/sjweh.4267

**Published:** 2026-03-01

**Authors:** Johannes Siegrist

**Affiliations:** 1 Institute of Medical Sociology, Centre for Health and Society, Faculty of Medicine, Heinrich-Heine-University Düsseldorf, Düsseldorf, Germany.

**Keywords:** depression, ERI, ischemic heart disease, psychosocial work environment, theoretical model

## Abstract

**Objective:**

This paper discusses the contribution of a widely used theoretical model of the psychosocial work environment, effort–reward imbalance (ERI), to occupational health research. It highlights the development of this approach, its measurement, and its main findings over the past three decades, focusing on epidemiological investigations. Furthermore, several limitations and challenges in view of far-reaching changes of modern work are discussed.

**Methods:**

Based on systematic reviews, meta-analyses, and an extended search for key publications, this discussion paper sets out the main evidence of associations of the model's measures with health risks, prioritizing prospective investigations. Complementing results addressing psychobiological markers as potential pathways underlying these associations, as well as findings on the model's expansion beyond paid work, are briefly summarized.

**Results:**

Currently available findings document consistent, moderately elevated related risks of ischemic heart disease (IHD) and depression following exposure to ERI. Quasi-experimental findings on physiological parameters as potential mediators of the link with IHD support this evidence. Results on a range of other disorders, in particular metabolic diseases, drug-related disorders, and indicators of reduced health functioning, while supportive, are less robust.

**Conclusions:**

This paper synthesizes three decades of international research on ERI as a parsimonious model of adverse psychosocial working conditions. At the same time, conceptual and methodological limitations—particularly in light of rapid changes in modern work and employment—point to priorities for future refinement and application of the model.

During the second half of the past century, the notion of psychosocial work environment as an occupational health risk received increased attention (1, 2). Swedish psychiatrist Lennart Levi, one of the early proponents, described it as an umbrella term for non-material work conditions that affect the health and well-being of workers (3). With rapid technological advances and major structural changes in modern economies, physically demanding work and exposure to noxious physical and chemical stressors, despite their continued significance, were no longer the dominant risks. Rather, a majority of modern jobs were characterized by psycho-mental and socio-emotional demands, often as sedentary work, and increasingly performed with digitalized devices. These work environments affect working people primarily through sensory input to the brain, triggering affective, cognitive and neurobiological responses, as analyzed in the framework of psychobiological stress research (4). Thus, occupational health scientists were confronted with the challenge of identifying health-related aspects within the complexity and variety of psychosocial work environments. To this end, theoretical models were proposed. A theoretical model in this domain is best described as a selection of distinct features of work environments, defined at a high level of abstraction, where interactions between these features, specified as research hypotheses, are expected to explain health outcomes. Importantly, these features are defined by terms that are open to transdisciplinary links between psychological and biological constructs as evidenced by stress research. Over the past 50 years, a number of such theoretical models have been proposed. Examples include 'person-environment fit' (5), demand–control' (or 'job strain') (6),'social support' (7),'organizational justice' (8), and 'effort-reward imbalance' (ERI) (9). More recently, several additional concepts emerged (for review, see 10), where 'job demands–resources' has been proposed as a unifying concept (11). These models focus on the organizational or workplace context and its relationships with working people, rather than on broader societal structures at the macro level. Some of these theoretical models, most importantly 'demand–control', have found wide application in international occupational health research, routine monitoring approaches, and even legislation (12). It is therefore of interest to review the contribution of these models to scientific advances. In this paper, given the rich body of empirical findings resulting from three decades of research, the focus is on ERI. In addition to reviewing main evidence, several conceptual and methodological challenges are discussed.

## Development and measurement of the effort-reward imbalance (ERI) model

The development of theoretical models is rarely the result of a sudden intellectual intuition but rather emerges from a critical appraisal of shortcomings of the current state of knowledge. In the case of the ERI, the development of the model involved a long exchange between empirical observations and theoretical reflection. It started with listening to the histories of young or middle-aged men who survived their first acute myocardial infarction, interviewed together with the authors' research team during their clinical rehabilitation. Many patients reported heightened strain in the months preceding disease onset. Guided by sociological concepts of social status and social identity, and informed by life event research (13), a rebcurring pattern was identified across accounts: an excessive investment in maintaining one’s primary social status—namely, the occupational role—paired with an abrupt threat to, or loss of, that role. To examine whether this pattern was specific to a group of men surviving their myocardial infarction, the team set up a case–control study, comparing the findings of this panel of 380 cardiac patients with those of a healthy control group, matched by age and sex and of similar size. Results demonstrated significantly more status-threatening life events, such as a 'broken promise' (eg, denied promotion by superior) or an unjustified downward shift (eg, moving a skilled worker to an unskilled job), compromising long-term rewards in the group of cardiac patients (14). A more precise assessment of indicators of psychosocial workload and threats to one's occupational status in the frame of a subsequent prospective study of an occupational high-risk group of blue-collar workers revealed that the co-manifestation of indicators of high effort and of low status-related reward was five times more frequent in the group with incident ischemic heart disease than in the remaining group (15). In line with the core sociological notion of reciprocity in social exchange and its violation (16), this finding laid the ground for proposing a novel theoretical notion of a health-adverse psychosocial work environment ERI (9).

With this new notion, main emphasis is put on the two essential elements of contractual employment-related exchange: efforts spent and rewards expected or received in turn at the three levels of financial (salary, wage), status-related (job security, promotion prospects) and socio-emotional transfer (esteem, appreciation). In line with exchange theory, balanced and unbalanced relationships are of main interest. As mentioned, unbalanced exchange, where loss matters more than gain (17), was considered the crucial trigger of working people's stressful experience. Thus, the core hypothesis asserts that conditions of high effort in combination with low reward act as main predictors of poor health. Although 'reward' as a core theoretical component is grounded in social exchange theory, it gives room to the notions of (job) security and equity/justice, which are explicit dimensions in complementary models of stressful work. An intrinsic element was added to these two extrinsic conditions, namely, the working person's way of coping with work-related demands. Here, a personal pattern of excessive coping, termed 'over-commitment', was identified. This risky pattern can result from the worker's biographical history (eg, as strong internalized motivation), or it can be evoked by environmental constraints (eg, piecework). Over-commitment is expected to act as an independent predictor of health and, in addition, may moderate associations of the two extrinsic elements with health.

To operationalize this theoretical concept, three scales were developed with Likert-scaled items. Effort and over-commitment were designated as unidimensional scales whereas reward was conceptualized as a second-order construct with the three respective subscales. Psychometric validation of the scales was performed, and the fit of the assumed theoretical structure of the scales with empirical data was examined by confirmatory factor analysis (18). This examination of the original questionnaire was repeated with a subsequent shorter version of the questionnaire (19). Notably, further tests of factorial invariance and stability over time corroborated the model's ability to assess responsiveness (20). Given a robust factorial structure of this measurement approach, the questionnaire has been translated in several languages, and respective psychometric quality criteria have been assessed, including confirmatory factor analysis (for review, see 21).

To test the model's main hypothesis of separate and combined effects of effort and reward on health, multivariable regression analyses were performed, where effects on health are analyzed for the single scales as well as for their combination. In this latter regard, most analyses were performed by additive interaction analysis of effort and reward or by application of an investigator- based algorithm, 'effort–reward ratio' (18). This latter procedure deserves a further comment. A quantification of this (im)balance, constructed by a ratio of the scale scores 'effort' (nominator) and 'reward' (denominator), which are weighted by scale item numbers, identifies a balanced exchange by values around 1.0, with lower scores indicating high gain with low cost and higher scores indicating high cost with low gain. As the relationship of the ratio with health was found to be non-linear, pointing to an elevated health effect only in scores >1.0, values in the upper tertile or quartile of the distribution were defined as exposure of interest. This algorithm is also of theoretical interest as it is thought to reflect a habituated persistent state of emotional unease resulting from a recurrently experienced unfavorable trade-off between expended effort and obtained reward at work (22). Evidently, high over-commitment can reinforce this effect.

According to the theory of affective information processing, repeated negative experience bypasses conscious awareness, eliciting an affective response in cortico-limbic circuits (23). Importantly, a transdisciplinary link integrates the model's key notion of reward with neuroscience information as stress-inducing sensory input is processed in the brain reward circuits and their connections with the main stress axes of the organism (24). For instance, experiencing failed reciprocity of exchange in terms of broken promises or violated trust evokes emotional pain responses in the insula, with strong visceral and somatic sensations (25). More generally, experimental neuroscience results revealed that the brain's reward circuitry is sensitive to the experience of disadvantageous inequality in social exchange (26). The model's additional hypothesis of a health effect of over-commitment was mainly tested by effect modification, but few epidemiologic studies extended their analysis beyond the main hypothesis.

Given the susceptibility to reporting bias, measuring this model with self-reported information obtained from answers to standardized questionnaire scales is subject to methodological criticism. This limitation has to be acknowledged, and efforts of bias control [eg, adjusting for personal traits (27), replacing individual scores by mean scores at the level of work units (28)] can only partly reduce this difficulty. Using a job exposure matrix was proposed as an alternative methodological approach (29), although a gain in objectivity goes along with a loss of precision of effect estimates. Within the methodological limitations, applying the questionnaire method has the advantage of providing more nuanced information whose validity is further strengthened by triangulation (eg, comparing observational with (quasi)-experimental data) (10).

## Empirical evidence

The ERI model has been widely tested in occupational health research, most often in observational epidemiological investigations, with a core interest in analyzing its contribution towards explaining elevated incident disease risks. Consequently, this discussion paper summarizes main findings from epidemiological research, preferring information from prospective cohort studies. However, the model's application goes far beyond this interest. As a first area of expanding research, in naturalistic or (quasi)-experimental studies, biological parameters are collected in combination with contextual data and subjective reports of participants. These studies are essential to inform scientists on psychobiological pathways underlying the associations of psychosocial exposures with disease occurrence. In this field of research, ERI was shown to be associated with elevated blood pressure (30), reduced heart rate variability (31, 32), altered cortisol secretion (33, 34), reduced immunity (35), and increased systemic inflammation (36, 37). This information supports the assumption that an adverse psychosocial work environment impacts directly on the development of stress-related disorders. A second area of expanded research concerns the application of the theoretical approach to the experience of failed reciprocity of exchange in unpaid social activities. So far, ERI has been associated with reduced health due to stressful household and family work (38), burden of caring (39), voluntary work (40), and educational work (41). Moreover, experiencing failed reciprocity jointly at work and in social activities was observed to increase the risk of cardiovascular mortality (42). In conclusion, this parsimonious theoretical notion has been applied to a broad spectrum of outcomes. This fact can be considered a strength of theoretical development (43).

Instead of extending the review to these two areas, as was the case in an earlier publication (44), this paper focuses on the main findings from occupational cohort studies. Its search strategy has been guided by the availability of several systematic reviews and meta-analyses, published between 2004 and 2025 and has been complemented by a systematic screening of relevant titles in main international journals, covering the past two decades. Given the author's continued involvement in research on this topic, a broad stream of information has been collected by an extended international network of informal scientific exchange. Concerning the selection criteria of included studies, articles had (i) be written in English language and published in a peer-reviewed journal, (ii) contain original or proxy measures of the ERI model, and (iii) provide quantified data on health, preferably adjusted for various observed confounders.

## Study findings in occupational epidemiology

Up to now, epidemiologic cohort studies have most frequently examined two chronic disorders: depression and ischemic heart disease (IHD). This prioritization reflects their high public health relevance, given a substantial contribution to global morbidity, mortality, and life expectancy (45). In summary, starting with an initial demonstration in the frame of the British Whitehall II study (27), the explanation of elevated IHD risk using the ERI model was analyzed in nine longitudinal studies (for review, see 46–48). In these investigations, the relative risk varied between 1.16 and 2.42, with an overall elevation of ~50% if compared to the risk of non-exposed participants. Two of these studies provided data on cardiovascular disease mortality. Some studies documented an effect modification by socioeconomic position, given a steep social gradient in the distribution of IHD (49). More recently, the joint exposure of ERI and job strain (the combination of high demand and low control at work) on IHD incidence was examined (48). In this study, among men, the relative IHD risk of the exposed group was twice as high as the risk of non-exposed workers and substantially higher than the risk of each single model. Additional indirect support of a link between stressful work and cardiovascular risk comes from studies testing associations of ERI with hypertension (30), atherogenic lipids (50), and incident Type 2 diabetes (51). Taken together, ERI at work is considered an independent psychosocial predictor of IHD, with an effect size comparable to that of job strain, the leading predictor in this domain (52).

**Figure 1:**
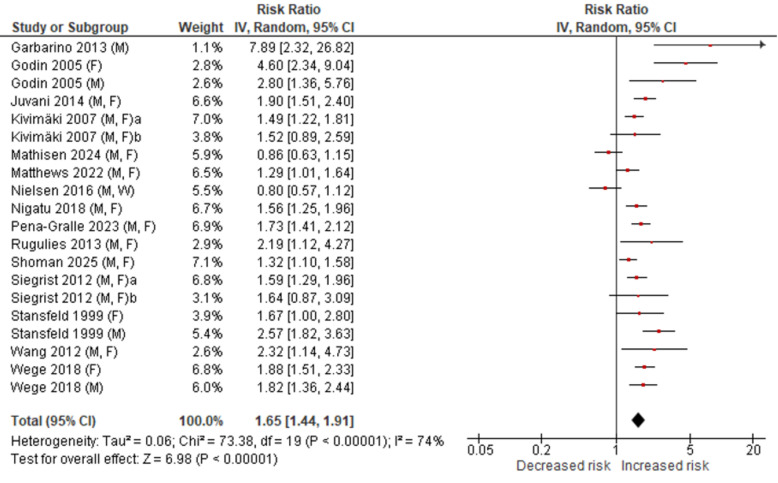
Meta-analysis of cohort studies on associations of effort-reward imbalance at work and depression. [F=Female; M=Male; CI=Confidence interval.] **Note:** Full references available in the supplementary material. Kivimäki 2007 (a) 10-town study; (b) Hospital Personnel study Siegrist 2012 (a) European study; (b) USA study.

The other main disease under study was depression, defined as clinically relevant depressive episode as assessed by validated measures. Figure 1 displays the results of a meta-analysis, based on findings of 20 reports from prospective investigations (47, 53, 54; for references see supplementary material, www.sjweh.fi/article/4267). Overall, the relative risk due to ERI was about 65% higher in the exposed groups, among both men and women. Despite wide confidence intervals in some studies, effects were statistically significant in a large majority of these reports. It should be noted that a multi-wave prospective study found stronger effects for short- compared to longer-term (2 versus 4–6 years) associations (55).

Again, the co-manifestation of ERI with job strain was explored. In a large Finnish study on disability pension due to depression, the risk of depression was more than four times as high in the group with joint exposure to ERI and job strain compared to the non-exposed group, and it was more than twice as high compared to the risk of those with only one exposure (28). Like IHD, the prevalence of depression follows a social gradient, with higher frequency among lower socioeconomic status groups. At least one prospective study demonstrated an expected effect modification by socioeconomic status, where the relative risk was highest among those who reported high scores on ERI and who belonged to the lowest occupational group (56).

Other health outcomes have been explored less often in prospective studies. Examples include reduced health functioning (49, 57) and in particular impaired cognitive functioning (58). Of interest, high reward at work was found to act as a protective cognitive resource (59, 60). Musculoskeletal pain, a highly prevalent limitation of physical functioning, was related to ERI in several reports (for review, see 61), with strong bi-directional associations (62). Moreover, ERI was found to be related to risky alcohol consumption (63, 64), and drug misuse (65, 66). More distant health-related variables with high economic and health policy impact were also studied in relation to ERI, in particular sickness absence (67) and turnover intentions (68). Yet, an important gap of knowledge needs to be mentioned, namely, the lack of results derived from intervention studies addressing the structural, organization-level adversities identified by the model. The few available methodologically sound intervention studies are instructed by a combination of elements derived from the ERI and the job strain model, and their documented health effects are restricted to burnout and prevalence of hypertension (69, 70). This lack of knowledge is not restricted to the ERI model, but reflects the methodological difficulties of applying best-practice intervention designs to the study of changes of psychosocial work environments. Although innovative approaches are desirable, an alternative way of strengthening evidence on causal links concerns the use of promising recent advances in statistical analyses of causal inference in observational studies (see below).

This selection of evidence illustrates the scope of applications of the ERI model in explaining health outcomes (box 1). Although respective effect estimates only rarely point to a doubling of risk, they are meaningful for preventive activities, addressing vulnerable populations. Combining ERI with job strain – the other most often studied established psychosocial predictor – results in a high burden of preventable diseases. Recent calculations of the population-attributable fractions estimated that ~23% of depressive episodes (71) and >19% of IHD events (72) among employed populations are attributable to this constellation. These estimates point to a relevant potential health gain resulting from related practical interventions.

Box 1 **Main messages**

- Conditions of high effort and low reward at work predict moderately elevated risks of several stress-related disorders.
- Evidence so far is most advanced for depression and coronary heart disease.
- Supplementary findings on indicators of potential psychobiologic pathways support the notion of a causal link.
- This new knowledge can instruct the development of theory-based interventions.

## Critical appraisal

This review of three decades of research on a theoretical model of stressful psychosocial work (ie, ERI) provides a synthesis of new knowledge on work-related determinants of health among employed populations. It illustrates the contribution of a parsimonious model to the prediction of disease onset and development, even after controlling for a complementary work stress model and for other relevant determinants (eg, age, sex, previous health). Despite this advantage, some inherent conceptual and methodological issues deserve further clarification, and a critical appraisal needs to address the model's limitations in view of a rapidly transforming world of work and employment.

Conceptually, this model in part overlaps with two related theoretical concepts: job strain and organizational justice. Importantly, effort is closely linked to the demand dimension of the job strain model. Yet, despite some operational communality, the theoretical interest of the two notions differs. Demand is analyzed as a component of job tasks, whereas effort reflects the perceived input required from the working person for expected return. Many studies tested and confirmed the independent explanatory contributions of the two models, in addition to their additive effects. Moreover, job strain is restricted to situational components, whereas the ERI model includes a personal component in terms of an individual coping characteristic. A second potential overlap concerns the dimension 'distributive justice' within the model of organizational justice. How is it linked to the notion of ERI? Distributive justice is defined as perceived equity/inequity of the organization's distribution of valuable goods or services among its members. Thus, a social comparison process among members offers the reference standard for justice judgments. In contrast, when effort is weighed against reward, it is the 'within-person comparison' which matters most, ie, the justice of exchange between 'give' and 'take' (73). This difference between external and internal standard of judgment is crucial. Again, some studies documented the independent contribution of the two models towards predicting health outcomes (74). Future studies might explore the explanatory contribution of combinations of these overlapping but separate models.

Two methodological problems concern the measurement of the model and applied statistical approaches of hypothesis testing. The majority of studies restricted their measurement to the extrinsic components of effort and reward, thus neglecting the inclusion of the model's intrinsic 'over-commitment' component. Moreover, replacing the original scales by proxy indicators in several studies reduced the comparability of findings. With extensive psychometric testing, the ERI scales were developed in the frame of classical test theory. This approach relies on some critical restrictions and may be complemented by a formative measurement model, including item–response theory (21). With regard to the main statistical approaches applied so far, an extension of established multivariable regression models towards a counterfactual approach to causal inference is desirable, specifically as it offers a more appropriate way of estimating mediation and moderation effects (75).

Finally, in view of the rapidly transforming world of work and employment, some limitations of this theoretical model are addressed. In times of economic globalization – with the large impact of transnational corporations and an extensive global flow of capital, trade, and workforce – enterprises, factories, and other organizations are strongly affected by macrostructural developments and constraints, limiting their autonomy and independence. The model's focus on the meso-social level does not adequately capture these aspects. The same holds true for the ground-breaking technological advances of automation, digitization and artificial intelligence that transform whole sectors of industrial and service work. In addition, national regulations of occupational safety and health closely shape company practices as firms are required to implement measures that aim at reducing psychosocial stress at work. For these reasons, theoretical models focusing on associations of psychosocial working conditions with the health of workers are expected to incorporate a multi-level approach, allowing the analysis of macrostructural determinants. In fact, with a focus on macrostructural conditions at the level of national labor and social policies, such an extension was already performed in the frame of ERI research. Findings from cross-national analyses demonstrate that active labor market policies exert a direct effect on mean levels of work stress in respective countries, and thereby, reduce the overall exposure to stressful conditions (for review, see 76). Moreover, as modern work and employment arrangements are increasingly characterized by flexibility, discontinuity, and fragmentation—including the growth of non-standard employment—exposure assessment should move beyond single time-point (mainly baseline) measurement toward dynamic models that capture entire employment histories. This challenge has already been tackled to some extent in the frame of ERI research by identifying several patterns of critical employment careers (77). As preliminary links with health indicators were observed, this extension can be considered a starting point of further conceptual and methodological developments of the model in the framework of life course research (78). Future developments along these lines are summarized in box 2.

Box 2 **Perspectives:**

Future developments along these lines should address important challenges:

- Extending exposure assessment to whole occupational careers.
- Including relevant macro-structural determinants into multi-level analysis.
- Integrating new work-non-work constellations, both conceptually and methodologically.
- Reconciling the parsimonious model with the need of tackling the complexity of modern work.

Closely linked to this limitation is the model's exclusive focus on work, thus neglecting substantial links between work and non-work in daily life. Flexible work, promoted by economic motivations, the expansion of digitalization, and the COVID-19 pandemic, has weakened the dominant role of stable workplaces as well as a rigid link of job performance with prescribed work schedules. Remote work and a series of new forms of work and non-work arrangements – including a shortening of working hours and weekly workdays – give more room to leisure time and activities that enrich or replace formal work. There are signs of a social reconstruction of the traditional work role and similarly of a diminished predominance of traditional collective values of work ethics, replaced by individual preferences. It will be important to investigate the extent to which a societal devaluation of the centrality of work in adult life reduces the burden of stressful work and its adverse effects on health. Thus, considering the integration of a multi-level and a life course approach, and in view of new types of work-non-work interactions, there is a need to apply tools of complexity analysis to this line of research (79)

In conclusion, this contribution illustrates the main achievements of three decades of occupational health research instructed by the concept of ERI. In view of a critical appraisal of this model, future developments are expected to reconcile the advantage of its parsimony with the need of tackling the complexity of modern work.

## Supplementary material

Supplementary material
