# Supplementary material for: Effort–reward imbalance at work and health: Review and critical appraisal of three decades of research
Source: Scand J Work Environ Health. 2026 Feb 28;52(2):179–88. doi: 10.5271/sjweh.4267 (PMC12966989; doi:10.5271/sjweh.4267)
Supplement: Supplementary material [file SJWEH-52-179-S001.pdf]

Effort–reward imbalance at work and health: Review and critical appraisal of three decades of research[1]

by Johannes Siegrist, PhD [2]

1. Supplementary material
2. Institute of Medical Sociology, Centre for Health and Society, Faculty of Medicine, Heinrich-Heine-University Düsseldorf, Düsseldorf, Germany.

References for Figure 1

Garbarino S, Cuomo G, Chiorri C, Magnavita N. Association of work-related stress with mental health problems in a special police force unit. BMJ Open 2013 3(7): e 002791. https://doi.org/10.1136/bmjopen-2013-002791.

Godin I, Kittel F, Coppieters Y, Siegrist J. A prospective study of cumulative job stress in relation to mental health. BMC Public Health 2005 15(5): 67. https://doi.org/ 10.1186/1471-2458-5-67.

Juvani A, Oksanen T, Salo P, Virtanen M, Kivimaki M, Pentti J, et al. Effort-reward imbalance as a risk factor for disability pension: the Finnish Public Sector Study. Scand J Work Environ Health 2014 40(3): 266–77. https://doi.org/ 10.5271/sjweh.3402.

Kivimäki M, Vahtera J, Elovainio M, Virtanen M, Siegrist J. Effort-reward imbalance, procedural injustice and relational injustice as psychosocial predictors of health: complementary or redundant models? Occup Environ Med 2007 Oct;64(10): 659-65. https://doi.org/10.1136/oem.2006.031310.

Mathisen J, Nguyen TL, Madsen IEH, Xu T, Jensen JH, Sørensen JK et al. Associations between psychosocial work environment factors and first-time and recurrent treatment for depression: a prospective cohort study of 24,226 employees. Epidemiol Psychiatr Sci. 2024 Mar 18;33: e13. https://doi.org/10.1017/S2045796024000167.

Matthews TA, Porter N, Siegrist J, Li J. Unrewarding work and major depressive episodes: cross-sectonal and prospective evidence from the US MIDUS study. J Psychiatry Res 2022 Dec:156: 722-728. https://doi.org/10.1016/j.jpsychires.2022.11.009.

Nielsen MB, Madsen IE, Aust B, Burr H, Rugulies R. Effort-reward imbalance at work and the risk of antidepressant treatment in the Danish workforce. J Affect Disord 2016 196: 248–51. https://doi.org/10.1016/j.jad.2016.02.038.

Nigatu YT, Wang J. The combined effects of job demand and control, effort-reward imbalance and work-family conflicts on the risk of major depressive episode: a 4-year longitudinal study. Occup Environ Med 2018 75(1): 6–11. https://doi.org/10.1136/oemed-2016-104114.

Pena Gralle APB, Talbot D, Trudel X, Milot A, Gilbert-Ouimet M, Lavigne-Robichaud M. et al. Socioeconomic inequalities, psychosocial stressors at work and physician-diagnosed depression: Time-to-event mediation analysis inthe presence of time-varying confounders. Plos one 2023 Oct; 18(10): e0293388. https://doi.org/10.1371/journal.pone.0293388.

Rugulies R, Aust B, Madsen IE. Effort-reward imbalance at work and risk of depressive disorders. A systematic review and meta-analysis of prospective cohort studies. Scand J Work Environ Health 2017 43(4): 294–306. https://doi.org/10.5271/sjweh.3632.

Shoman Y, Ranjbar S, Strippoli MF, von Känel R, Preisig M, Guseva Canu I. Longitudinal association of exposure to work-related stress with major depressive disorder and the role of occupational burnout in this association in the general population. Soc Psychiatry Psychiatr Epidemiol. 2025 Mar;60(3): 593-606. https://doi.org/10.1007/s00127-024-02735-w.

Siegrist J, Lunau T, Wahrendorf M, Dragano N. Depressive symptoms and psychosocial stress at work among older employees in three continents. Glob Health 2012 8: 27. https://doi.org/10.1186/1744-8603-8-27.

Stansfeld SA, Fuhrer R, Shipley MJ, Marmot MG. Work characteristics predict psychiatric disorder: prospective results from the Whitehall II study. Occup Environ Med 1999 56(5): 302–307. https://doi.org/10.1136/oem.56.5.302.

Wang J, Patten SB, Currie S, Sareen J, Schmitz N. A population-based longitudinal study on work environmental factors and the risk of major depressive disorder. Am J Epidemiol 2012 176 (1): 52–59. https://doi.org/10.1093/aje/kwr473.

Wege N, Li J, Siegrist J. Are there gender differences in associations of effort-reward imbalance at work with self-reported doctor-diagnosed depression? Prospective evidence from the German Socio-Economic Panel. Int Arch Occup Environ Health 2018 91(4): 435–43. https://doi.org/10.1007/s00420-018-1293-8
